# Supplementary material for: Nutritional Conditions Modulate C. neoformans Extracellular Vesicles’ Capacity to Elicit Host Immune Response
Source: Microorganisms. 2020 Nov 18;8(11):1815. doi: 10.3390/microorganisms8111815 (PMC7698703; doi:10.3390/microorganisms8111815)
Supplement: Supplementary file 1 [file microorganisms-08-01815-s001.pdf]

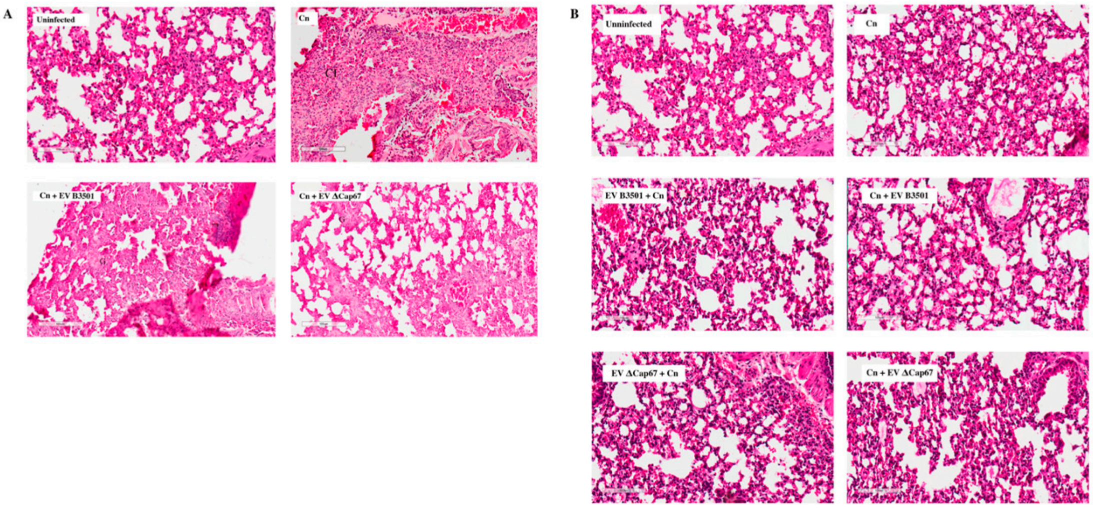

Figure S1. Treatment of infected mice with EVs reduced inflammation and cellular infiltration in lungs with 5 dpi. C57Bl/6 mice were infected with *C. neoformans* (2×10^4 cells/animal) intratracheally and treated intranasally with 50 µg of EVs (protein content) produced in SDB 100%. Mice were treated one and three dpi and euthanized at 5 dpi (**A**) or treated two and four dpi and euthanized at 15 dpi (**B**). Lungs were removed, and fragments were separated for hematoxylin-eosin stain and histopathological analysis. In figure: G: granulomas; CI: cellular infiltrate. Magnification of 40× and bar represents 100 µm.
